# Timing of primary maxillary tooth eruption in preterm compared to term infants: a retrospective longitudinal cohort study

**DOI:** 10.1186/s12903-026-08851-0

**Published:** 2026-06-16

**Authors:** Ariane Hohoff, Linda Bosse, Thomas Stamm, Jonas Q. Schmid, Moritz Kanemeier

**Affiliations:** Department of Orthodontics, University of Münster, Albert-Schweitzer-Campus 1, Gebäude W 30, Münster, 48149 Germany

**Keywords:** Preterm, Term, Tooth eruption

## Abstract

**Background:**

Reported eruption times of primary teeth in term and preterm infants are largely based on detection of visible tooth structure at the gingival level by parents, paediatricians, or dentists. Furthermore, there is a lack of quantitative data on primary tooth eruption velocity in the literature. This study aimed to compare eruption timing, eruption velocity, and maximum crown length by modelling longitudinal crown eruption trajectories in term and preterm infants.

**Methods:**

This retrospective longitudinal cohort study included 100 infants (51 preterm, 49 full-term) who were followed from the first weeks after birth with quarterly assessments over four years. A total of 626 serial maxillary plaster casts were obtained as part of a prospective clinical trial registered at clinicaltrials.gov (NCT00408746). The crown length of 1,885 primary teeth (central and lateral incisors, canines, first molars) was measured by one investigator on digitised casts using Blender software. Intrarater reliability was evaluated using intraclass correlation coefficients (ICC). Timing and velocity of eruption were estimated using a non-linear asymptotic growth model.

**Results:**

Measurement reliability was excellent (ICC = 0.996). Comparing chronological age revealed that preterm infants had a mean eruption delay of 2.7 months compared to full-term infants, with a significant delay observed for canines (4.5 months). However, when corrected age (chronological age minus days born before 37 gestational weeks) was used, a mean delay of only 1.4 months with no significant differences was found. Model-derived eruption preceded commonly reported clinical eruption ages by 6.2 months in preterm and 7.7 months in full-term infants. The model-estimated maximum crown lengths were slightly smaller in preterm infants, though these differences were not statistically significant. Eruption velocity followed a non-linear pattern, with preterm infants showing a tendency towards higher eruption rates, suggesting catch-up growth.

**Conclusions:**

Based on our model, eruption timings reported in the literature likely correspond to clinical observations made when primary teeth have attained about half of their final crown height. Consequently, crown exposure through gingiva appears to occur on average about six months prior to clinically visible eruption. Although preterm infants show a delay in tooth eruption when assessed by their chronological age, this difference resolves after correction for prematurity.

## Background

Primary tooth eruption is an important developmental milestone during infancy. Because the appearance of the first teeth influences feeding behaviour, oral hygiene practices, and parental concerns, reliable information on the timing and pattern of eruption has practical relevance for counselling in pediatrics and dentistry. This is particularly important for infants born preterm, whose early growth and maturation trajectories may differ from those of term infants.

Preterm birth, defined as delivery prior to 37 completed weeks of gestation, is categorised by the World Health Organization into three gestational-age (GA) groups: extremely preterm (GA < 28 weeks), very preterm (28 $\le$ GA < 32 weeks), and moderate-to-late preterm (32 $\le$ GA < 37 weeks) [1].

Preterm infants are particularly susceptible to a range of complications owing to the immaturity of their physiological systems [2, 3], and although advances in medical care have markedly improved survival rates, these gains have been accompanied by an increased incidence of serious morbidities, emphasising the need for specialised medical and dental care for this group [2]. Preventive measures can only be implemented once the problems arising from premature birth have been precisely characterised.

The orofacial region is crucial for infant development, as this region plays an essential role in breathing, feeding, speaking and social interaction. From the onset of oral function until the establishment of full oral feeding in the neonatal period, a complex interaction of internal and external factors can affect orofacial, and in particular, palatal development [4–6]. In the early stages of oral formation the palatal bones are soft and highly malleable, so external pressure can readily alter palatal morphology. Consequently, the palate is particularly susceptible at this stage to influences that may affect dental development and facial appearance [4].

Oral complications commonly observed in preterm infants include alveolar ridge notching, palatal grooving, a high-arched palate, dental crossbite, and palatal asymmetry which may contribute to increased orthodontic treatment need. Furthermore, an increased risk of Angle Class II malocclusion and deep bite has been documented in this population [7–9].

Multiple studies have also reported a high prevalence of developmental defects of enamel (without higher prevalence of caries) and of tooth shape anomalies in children born at lower gestational ages, including reduced tooth-crown dimensions in permanent incisors and first molars [3, 10, 11].

The timing of the eruption of primary teeth serves as a visible sign of physical growth in infants that goes beyond body weight or height and has already been correlated with many stages of development [12]. This parameter also reflects neurological integrity and underlying medical conditions and is therefore a matter of substantial concern for parents during health evaluations [13]. Preterm infants commonly show developmental delays, including later eruption of primary teeth relative to their chronological age [13]. When timing is referenced to corrected age (i.e. chronological age minus the days of birth prior to the completion of the 37th week of gestation), however, eruption ages are either earlier [14] or within normal limits [15–17].

Evidence from the international literature indicates that the mean age of primary tooth eruption has declined over recent decades, particularly in economically advantaged regions [13]. It is therefore necessary to ascertain whether this trend is continuing, as has been observed for other somatic developments [18], or has now stabilised. At the same time, most eruption data are based on parental or clinical detection of visible tooth structure at the gingival level, which may represent a relatively late stage of the eruption process and may not capture early emergence dynamics. Serial three-dimensional models allow eruption to be quantified as a continuous process by measuring crown exposure over time.

Accordingly, this study aimed to compare eruption timing as the primary outcome, and eruption velocity and maximum crown length as secondary outcomes, by modelling longitudinal crown eruption trajectories in term and preterm infants. We hypothesised that preterm infants would show delayed eruption when assessed by chronological age, but that group differences would diminish when corrected age is used. We additionally hypothesised that eruption velocity differs between preterm and term infants.

## Methods

The current study is a retrospective longitudinal cohort study of infants enrolled in a clinical trial registered under clinicaltrials.gov (NCT00408746). The inclusion criteria of the original clinical trial were caucasian origin and written informed consent from both parents for all infants. For term infants, additional inclusion criteria were birth at $\ge 37$ weeks of gestation and birth weight $\ge 2500$ g. For preterm infants, additional inclusion criteria were gestational age at birth $> 25$ weeks and birth weight $> 500$ g. The exclusion criteria were hydrocephalus, oral or facial clefts, congenital syndromes, deformity of the head and neck, and congenital metabolic disease other than osteopenia of prematurity. No additional inclusion or exclusion criteria were applied for the present analysis. Plaster casts were made from upper jaw alginate (Blue Print Xcreme Alginate, Dentsply) impressions taken from preterm and term infants born at the tertiary-care University Hospital of Münster, Germany. Impressions were intended to be taken within the first week after birth when clinically feasible and subsequently at various examination times over the course of several quarters.

**Fig. 1:**
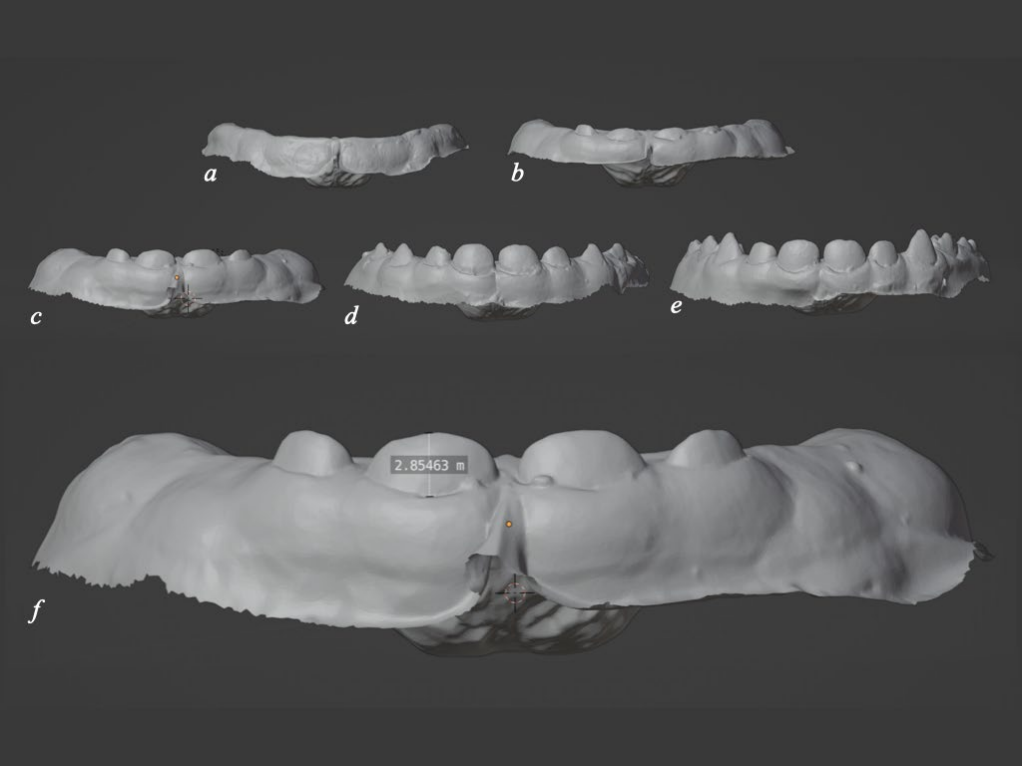
Sequence of jaw models of a premature infant to determine the eruption times. For measurement purposes, the digital models are aligned with regard to orthodontic diagnostics, with the alveolar process and dental arch facing upwards. **a** at four months of age, **b** at 11 months of age, **c** at 13 months of age, **d** at 20 months of age, and **e** at 28 months of age. The Blender’s measure tool was used to measure the length of the erupted tooth crown (**f**)

The dental casts were digitised using the ATOS II system (GOM GmbH, Braunschweig, Germany) and stored in the standard tessellation language file format (STL), for further processing. The STL files were imported into Blender 3.6.1 (Blender Foundation, blender.org) and symmetrically aligned to the grid floor (zero height) in Blender’s world coordinate system. Crown lengths in the gingival–coronal direction were measured using the “Measure” tool along the long axis of each erupted tooth crown (Fig. 1).

The measurement error was evaluated based on the intraindividual reproducibility of repeated measurements. For this reason, ten randomly selected models were measured twice at intervals of 10 days by investigator LB.

### Statistics

The statistical analysis of the data was carried out using R (version 4.5.2 [19]). Intrarater reliability was assessed using an intraclass correlation coefficient (ICC) based on a two-way mixed-effects model for absolute agreement (McGraw and Wong [20]) implemented in the irr package [21]. The level of reliability was defined according to Koo and Li [22]: poor reliability $<0.5$, moderate reliability $<0.75$, good reliability $<0.9$, excellent reliability $>0.9$.

#### Tooth eruption

The primary outcome was the timing of tooth eruption ($t_0$), defined as a model-based age at which the crown length equals 0 mm. Secondary outcomes were the maximum crown length (*A*) and the rate of crown eruption (*k*). The main comparison was between preterm and full-term infants. Longitudinal crown length measurements were analysed using a non-linear asymptotic model, which uses repeated longitudinal measurements to estimate a population-level eruption curve. Clinically, differences in $t_0$ reflect earlier or later tooth eruption, while *A* and *k* describe the extent and velocity of crown eruption.

Specifically, the relationship between crown length and patient age (for preterm infants using both chronological and corrected age) was modelled as:

$$\begin{aligned} \text {length} = A - A \; e^{k (t_0 - \text {age})} \end{aligned}$$

Here, *A* represents the maximum attainable crown length, *k* the growth rate, and $t_0$ the estimated age at eruption. In the present model, eruption is defined as the age at which the crown length equals zero. Thus, when $\text {age}=t_0$, the exponential term equals one and the predicted crown length is $A-A=0$. Accordingly, $t_0$ represents the model-based zero-length intercept of the eruption curve, i.e. the back-extrapolated onset of crown emergence, rather than a clinically recorded eruption date. Age was modelled in days (therefore, *k* is expressed in $1/\text {day}$); for readability, eruption times are reported in months in the results. For preterm infants, analyses were performed using both chronological age and corrected age (corrected age = chronological age minus the number of days the infant was born prior to the completion of the 37th week of gestation).

Separate models were fitted for each combination of the primary tooth types included (central incisor, lateral incisor, canine and first molar) and the birth group (full-term and preterm). Parameter estimation was performed using non-linear least squares. To obtain robust estimates of uncertainty, 1000 non-parametric bootstrap replicates were drawn from each dataset, and 95% confidence intervals were derived for each model parameter (*A*, *k*, $t_0$) as well as for the differences in parameter estimates between preterm and full-term groups. Group differences were quantified as preterm minus full-term parameter estimates. To account for multiple comparisons across tooth types and model parameters, *p* values were adjusted using the Benjamini–Hochberg method.

## Results

One hundred infants were included in the study, of whom 51 were preterm (22 females, 29 males) and 49 were full-term (30 females, 19 males). The preterm group was significantly younger, shorter, had lower body weight, and also had a significantly smaller head circumference at the time of the first examination ($p<0.001$) than the term group (Table 1). The first examination, which included an alginate impression of the maxilla, was performed at a mean of $10.7 \pm 16.0$ weeks after birth in the preterm group and at a mean of $2.4 \pm 2.8$ weeks after birth in the term group. The corrected age at the first examination for the preterm group was $5.1 \pm 15.5$ weeks.

**Table 1:** Characteristics of the study groups at birth and at the day of the first orthodontic examination. Values presented as mean ± standard deviation

Parameter	Preterm	Term	*p*-value
Birth			
Gestational age [weeks]	31.4 ± 3.3	39.1 ± 1.3	< 0.001
Weight [g]	1460 ± 568	3512 ± 501	< 0.001
Length [cm]	38.4 ± 5.2	50.9 ± 2.5	< 0.001
Head circumference [cm]	27.2 ± 3.5	35.0 ± 1.2	< 0.001
First examination			
Weeks after birth	10.7 ± 16.0	2.4 ± 2.8	< 0.001

The average study period was 196.2 weeks (SD = 95.3, range = 48.7 - 306.6) for all patients, 186.7 weeks (SD = 93.4, range = 52.7 - 301.7) for preterm patients, and 206.2 weeks (SD = 97.1, range = 48.7 - 306.6) for full-term patients. The final impression was obtained 193.8 ± 94.0 weeks after birth (188.2 ± 93.0 weeks when corrected for prematurity) in the preterm group and 208.6 ± 96.4 weeks after birth in the term group. Overall, 626 impressions of upper jaws were available for analysis, of which 368 impressions had erupted teeth present. The mandibles were excluded from this study because lower jaw impressions were not taken at the start of the study. Impressions of both jaws became feasible only at a later age.

A total of 2,299 teeth were assessed, of which 229 were excluded because they were insufficiently represented on the plaster models. Owing to the late eruption of the second primary molar and the first permanent molar, there was insufficient data (n = 185) for these teeth, and they were therefore excluded. Thus, deciduous crown heights of a total of 1,885 teeth were measured and included in the final analysis.

The repeated measurement of the crown heights of 10 cases by LB resulted in a total of 52 repeated measurements. The ICC for these measurements was 0.996 suggesting an excellent intrarater reliability.

### Time of eruption

**Fig. 2:**
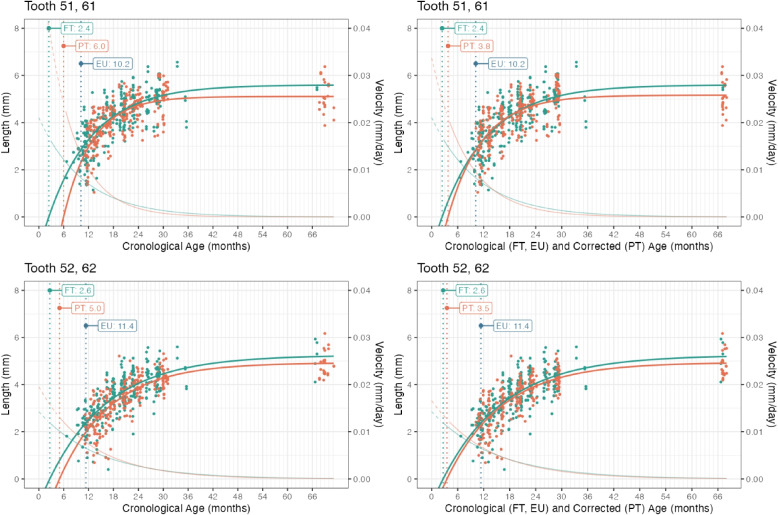
Crown length (thick lines) and velocity (thin lines) estimated by the non-linear model, along with the actual measurements (points), for full-term (green) and preterm (red) patients across anterior teeth. Velocity lines are dashed for the period prior to the estimated eruption. Vertical dotted lines indicate the estimated tooth eruption for full-term (FT; green) and preterm (PT; red) patients, as well as eruption timings reported for children born in Europe [23] (EU; blue). Age for preterm patients is shown chronological on the left and corrected for prematurity on the right

**Fig. 3:**
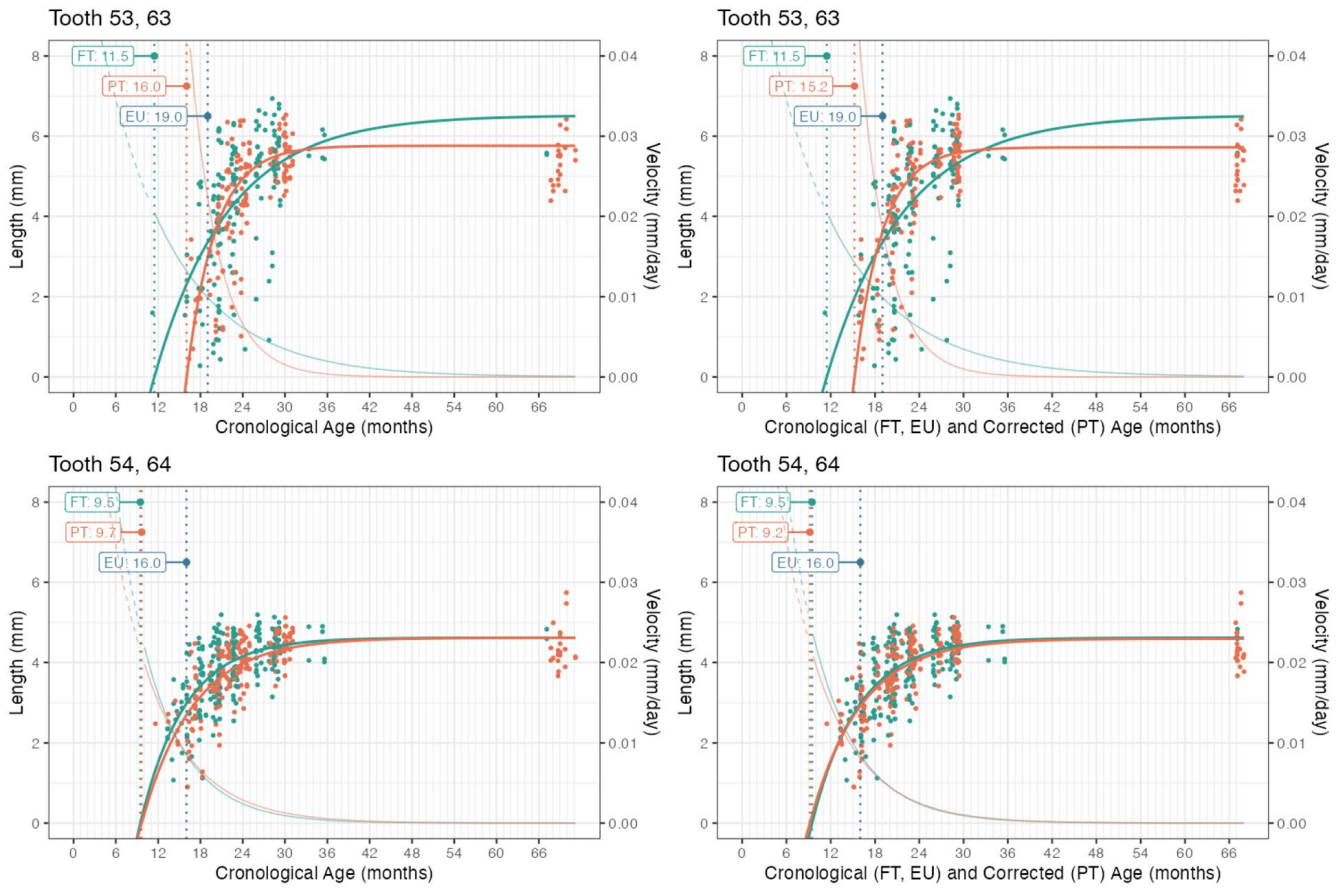
Crown length (thick lines) and velocity (thin lines) estimated by the non-linear model, along with the actual measurements (points), for full-term (green) and preterm (red) patients across posterior teeth. Velocity lines are dashed for the period prior to the estimated eruption. Vertical dotted lines indicate the estimated tooth eruption for full-term (FT; green) and preterm (PT; red) patients, as well as eruption timings reported for children born in Europe [23] (EU; blue). Age for preterm patients is shown chronological on the left and corrected for prematurity on the right

The estimated non-linear model derived from individual crown length measurements shows a typical progression of empirical growth trajectories and enables a theoretical estimation of the eruption time (Table 2; Figs. 2, 3). Intersections of the curves with the x-axis indicate the estimated model-derived eruption times ($t_0$).

**Table 2:** Estimates for the non-linear asymptotic model: Chronological age at eruption ($t_0$, month), maximum attainable crown length (*A*, mm), and growth rate constant (*k*, 1/day). Ages of preterm infants are reported both chronologicaly and corrected for prematurity

	Teeth	*t*_0_	(M [95% CI])	*A*	(M [95% CI])	*k*	(M [95% CI])
Full-term							
	51, 61	2.4	[0.4, 4.0]	5.60	[5.25, 6.17]	0.0030	[0.0022, 0.0040]
	52, 62	2.6	[0.3, 4.5]	5.25	[4.83, 5.81]	0.0023	[0.0017, 0.0031]
	53, 63	11.5	[9.0, 14.4]	6.52	[5.72, 8.65]	0.0031	[0.0017, 0.0058]
	54, 64	9.5	[7.4, 11.1]	4.62	[4.41, 4.89]	0.0052	[0.0037, 0.0071]
Preterm (chronological age)							
	51, 61	6.0	[3.3, 7.9]	5.11	[4.90, 5.37]	0.0047	[0.0034, 0.0066]
	52, 62	5.0	[3.2, 6.5]	4.93	[4.71, 5.16]	0.0027	[0.0022, 0.0032]
	53, 63	16.0	[14.8, 16.9]	5.76	[5.49, 6.34]	0.0080	[0.0056, 0.0110]
	54, 64	9.7	[7.9, 11.8]	4.61	[4.45, 4.79]	0.0045	[0.0037, 0.0060]
Preterm (corrected age)							
	51, 61	3.8	[0.9, 6.0]	5.17	[4.95, 5.40]	0.0041	[0.0030, 0.0057]
	52, 62	3.5	[1.7, 5.0]	4.94	[4.73, 5.19]	0.0026	[0.0021, 0.0031]
	53, 63	15.2	[14.2, 15.9]	5.72	[5.48, 6.26]	0.0087	[0.0059, 0.0120]
	54, 64	9.2	[7.6, 10.9]	4.59	[4.44, 4.78]	0.0048	[0.0040, 0.0060]

When chronological age is considered for both preterm and full-term patients, a discrepancy of $t_0$ is apparent between the groups (Figs. 2, 3, left; Table 3) with a mean difference of 2.7 months and when the corrected age was used a mean delay of only 1.4 month was found (Figs. 2, 3, right; Table 3). The differences were only significant for canines when considering the chronogical age.

**Table 3:** Differences in parameter estimates between preterm and full-term infants from the non-linear asymptotic model: Chronological age at eruption ($t_0$, month), maximum attainable crown length (*A*, mm), and growth rate constant (*k*, 1/day). Ages of preterm infants are reported both chronologicaly and corrected for prematurity. Positive differences indicate a higher value in preterm infants. Reported *p*-values were adjusted for multiple comparisons across tooth types and model parameters using the Benjamini–Hochberg method

Teeth	*t*_0_	(M [95% CI])	*p*_*adj*_	*A*	(M [95% CI])	*p*_*adj*_	*k*	(M [95% CI])	*p*_*adj*_
Difference between preterm (chronological age) and full-term									
51, 61	3.6	[ 0.3, 6.5]	0.072	-0.49	[-1.05, -0.05]	0.072	0.0017	[ 0.0000, 0.0040]	0.115
52, 62	2.3	[-0.3, 4.9]	0.168	-0.32	[-0.93, 0.17]	0.300	0.0004	[-0.0005, 0.0012]	0.552
53, 63	4.6	[ 1.0, 7.1]	0.040*	-0.76	[-2.75, 0.30]	0.289	0.0049	[ 0.0008, 0.0078]	0.040*
54, 64	0.2	[-2.3, 3.5]	0.890	-0.00	[-0.32, 0.29]	0.958	-0.0007	[-0.0028, 0.0016]	0.701
Difference between preterm (corrected age) and full-term									
51, 61	1.4	[-2.2, 4.5]	0.737	-0.44	[-0.99, 0.01]	0.224	0.0011	[-0.0004, 0.0030]	0.389
52, 62	0.9	[-2.0, 3.5]	0.763	-0.31	[-0.92, 0.19]	0.452	0.0003	[-0.0006, 0.0011]	0.763
53, 63	3.8	[ 0.3, 6.3]	0.169	-0.80	[-2.79, 0.22]	0.389	0.0056	[ 0.0014, 0.0089]	0.052
54, 64	-0.3	[-2.6, 2.5]	0.846	-0.02	[-0.34, 0.27]	0.846	-0.0004	[-0.0026, 0.0017]	0.846

*p < 0.05

The first measurements that could be performed on the study models (left-most points in Figs. 2, 3), thus representing the first visible tooth structures, are consistent with clinical studies conducted on children born in Europe [23]. This concurrence supports our mathematical model and suggests that initial gingival penetration of a deciduous tooth may occur approximately 6.2 months before clinical visibility in preterm infants (refering to age corrected for prematurity) and approximately 7.7 months before clinical visibility in full-term infants (Table 4).

**Table 4:** Differences in tooth eruption between literature values for infants born in Europe [23] and model-based estimates for full-term and preterm infants. All ages are reported in months, ages of preterm infants are reported chronologically and corrected for prematurity

Teeth	Europe	Model	Europe - Model
Full-term			
51, 61	10.2	2.40	7.8
52, 62	11.4	2.63	8.7
53, 63	19.0	11.5	7.6
54, 64	16.0	9.47	6.6
Mean	14.1	6.49	7.7
Preterm (chronological age)			
51, 61	10.2	5.97	4.2
52, 62	11.4	4.98	6.4
53, 63	19.0	16.0	3.0
54, 64	16.0	9.66	6.4
Mean	14.1	9.16	5.0
Preterm (corrected age)			
51, 61	10.2	3.76	6.4
52, 62	11.4	3.52	7.8
53, 63	19.0	15.2	3.8
54, 64	16.0	9.17	6.9
Mean	14.1	7.92	6.2

### Maximum crown length

The estimated maximum crown lengths were smaller in preterm than in full-term infants, but these differences were not statistically significant. The pattern of tooth size was consistent across the groups: the canine exhibited the greatest crown length, followed by the central incisor, the lateral incisor and the first molars.

### Velocity of eruption

With the exception of the first molar, preterm infants showed a generally higher growth rate constant. However, only for the canines a statistically significant difference in growth rate was found in the model using the chronological age for preterm infants.

The eruption velocity (millimetres per day) was calculated from the growth rate constant for both groups with respect to chronological age, and with respect to corrected age for preterm patients (Figs. 2, 3, light lines). Exact velocities were obtained from the model at $t_0$ in the preterm and full-term models, and at the mean European [23] $t_0$ values. In addition, age and eruption velocity at the point of half-eruption (i.e. half of maximum attainable crown height *A*) were calculated. The respective values are presented in Table 5.

**Table 5:** Model estimated velocities of primary teeth ($\mu$m/day) at three different time points: the model-estimated time of eruption (Model$t_0$), the model-estimated time at which half of the maximum crown length is reached (Model$t_{half}$), and literature-reported eruption time (Europe$t_0$). All ages are reported in months, ages of preterm infants are corrected for prematurity (birth before completion of the 37th gestational week)

	Teeth	Model *t*_0_		Model *t*_half_		Europe *t*_0_		M. *t*_half_$\rightarrow$E. *t*_0_	
		Age	Velocity	Age	Velocity	Age	Velocity	Age	Velocity
Full-term									
	51, 61	2.4	16.9	9.9	8.4	10.2	8.3	0.3	-0.1
	52, 62	2.6	11.9	12.7	5.9	11.4	6.5	-1.3	0.6
	53, 63	11.5	20.5	18.7	10.2	19.0	9.9	0.3	-0.3
	54, 64	9.5	24.0	13.8	12.0	16.0	8.5	2.2	-3.5
Preterm (chronological age)									
	51, 61	6.0	24.1	10.8	12.1	10.2	13.2	-0.6	1.2
	52, 62	5.0	13.1	13.6	6.5	11.4	7.8	-2.2	2.3
	53, 63	16.0	46.2	18.9	23.1	19.0	22.2	0.1	-0.9
	54, 64	9.7	20.9	14.7	10.4	16.0	8.7	1.3	-1.7
Preterm (corrected age)									
	51, 61	3.8	21.1	9.3	10.5	10.2	9.5	0.9	-1.0
	52, 62	3.5	12.6	12.4	6.3	11.4	6.9	-1.0	0.6
	53, 63	15.2	49.9	17.8	24.9	19.0	18.2	1.2	-6.7
	54, 64	9.2	22.2	13.9	11.1	16.0	8.1	2.1	-3.0

It could be shown that, except for the primary first molars, the eruption velocity in the preterm group was higher than in the term group at the time of eruption, and that this difference persisted following further tooth eruption. We hypothesise that this pattern is indicative of catch-up growth.

When age and the rate of eruption are considered at the point when the teeth are approximately half-erupted, values show remarkable agreement with those reported in the literature [23] both for preterm and for term infants. Literature-reported eruption times range from 1.3 months earlier (full-term lateral incisors) to 2.2 months later (full-term first molars) than the time of half-eruption predicted in our study, with a mean difference of 0.6 months after half-eruption (Table 5). It can therefore be assumed that in most studies the clinical assessments of tooth eruption were made when the teeth were already substantially erupted.

## Discussion

To our best knowledge, this is the first study that assessed tooth eruption on digitized three dimensional plaster models to estimate eruption time and eruption velocity of primary teeth in a group of preterm and full-term infants. Current literature comprises mainly clinical assessments; these pertain solely to the visible tooth substance and therefore cannot capture the initial emergence of a tooth through the gingival tissue.

### Time of eruption

Chronological eruption timings differ by continents [23]: Literature-reported mean eruption ages for maxillary primary teeth were lowest in Europe (8.2 months), followed by North America (9.4 months), Africa (9.9 months), Oceania (10.8 months) and Asia (11.1 months).

Another factor which is most likely related to the socio-economic status of the different continents, is birth weight, which is positively associated with earlier eruption of the first tooth. A difference of up to 4.2 months has been reported between infants born weighing less than 1500 g and those weighing more than 2500 g [24]. Use of infant formula and the resulting accelerated weight gain has a similar effect. Greater velocity of weight gain during the first 4.5 months was associated with earlier eruption of the first tooth [25].

Therefore, nutritional status must be considered an essential factor when comparing cohorts of preterm and full-term infants. Preterm infants demonstrate delayed tooth eruption when age is assesed chronologicaly; however, when developmental progress is assessed using corrected age no differences are evident between preterm and term infants [14–16]. This association is also confirmed by our findings.

In addition, the close agreement between literature-reported eruption ages and the model-estimated time point at which teeth reached approximately half of their asymptotic crown height *A* suggests that many clinical eruption reports may correspond to a mid-eruption stage rather than the initial eruption. This interpretation is consistent with the fact that clinical assessments typically record the first clearly visible tooth substance and are limited by examination intervals.

### Maximum crown length

Although not statistically significant, the estimated maximum crown length in our preterm group were consistently lower than those of the full-term group. Possible explanations of different measurements include gingival thickness or enamel defects. Tooth crown height measurements are limited in the literature, the majority of studies focus on horizontal tooth dimensions, developmental defects of enamel (hypoplasia, opacity) and dental caries as well as on the effect of early dietary mineral intake on enamel development [26–31].

Backström et al. [32] assessed the eruption of primary teeth and did not observe delayed maturation in preterm infants (using age corrected for prematurity) compared with a control group. They also found no effect of vitamin D supplementation during the neonatal period. Although the authors assessed only the occurrence of eruption, irrespective of the extent of crown emergence, these findings are consistent with ours.

Findings on horizontal tooth dimensions in the primary dentition are inconsistent. Earlier studies reported smaller mesiodistal and labiolingual tooth dimensions in preterm infants [26, 27, 30], but a later study did not confirm this [31]. Because the present study recorded only vertical measurements, comparisons must rely on percentage differences between full-term and preterm infants reported elsewhere: 6–11% for incisors [30] and approx. 3% for canines and molars whereas canines showed the greatest difference [28]. The results correspond with our model estimates, indicating that preterm central incisors are 7.7% and lateral incisors 5.9% shorter, with the greatest difference observed in canines (12.3%) and a negligible difference in first molars (0.6%). However, none of these differences were statistically significant.

Preterm infants have predominantly hypoplasias in the anterior upper primary teeth compared to term infants which is independent of calcium, phosphorus and vitamin D intake [29]. A possible explanation for the shorter crown lengths observed in our study is that micro-abrasion of these hypoplasias may have reduced crown height, particularly when measuring the greatest crown length in teeth subjected to the longest period of use.

### Velocity of eruption

Preterm infants showed, with the exception of the first molar, a generally higher eruption velocity both at the onset of eruption and at the point of model-estimated half-eruption, both for chronological and corrected age. Since the difference in growth rate constants was statistically significant only for the canines, these results should be interpreted as a trend only. We speculate that this trend is indicative for catch-up growth.

To the best of our knowledge, eruption velocity for deciduous teeth is not reported in the literature. To assess how realistic the eruption rates produced by our model are, it therefore seems appropriate to draw on biologically analogous growth rates. It can be assumed that there is a relationship between the rate of tooth eruption and root formation, since beyond a certain stage of dental development both processes proceed in parallel.

Massler and Schour [33] previously described a growth gradient in enamel and dentine formation that decreases with advancing age of the ameloblasts and odontoblasts. For primary teeth they calculated mean growth rates (incisor, canine, first molar) of 5.5–8.0 $\mu$m/day.

Dean and Vesey [34] calculated root growth rates at time points corresponding to different root lengths. At the onset of root growth the rate was 4 $\mu$m/day, increasing to 12 $\mu$m/day by the end of root formation. For canines they observed an initial acceleration, followed by a deceleration and then a subsequent catch-up. Our values lie within this range and likewise exhibit a non-linear trajectory, which lends support to our model.

Irurita Olivares et al. [35] built a model to estimate age from the length of deciduous teeth for forensic purposes. By rearranging their formula to predict the length at a given age, and by comparing the predicted age for consecutive days, an estimate of the velocity established by their data can be calculated. Using this method the velocities for the time of eruption reported in the literature [23] were calculated. The velocities were in the range of 9–14 $\mu$m, which is comparable to the range in velocities calculated using our model for the same time points (8-18 $\mu$m; Table 5).

In summary, owing to the lack of published data on eruption rates of primary teeth, no conclusive statement can be made about our model. Nevertheless, the rates we calculated closely mirror the published biological growth patterns of dental hard tissue formation [33, 34]. Furthermore, our data support the theories of dentition which propose that tooth eruption is not linear, but rather that teeth erupt with distinct, stage-specific rates [36].

### Limitations

Limiting the focus to the maxilla represents only one aspect of overall oral development. However, the maxilla is a critical structure for feeding, for speech, for dentition and for overall orofacial development, and is particularly sensitive to exogenous influences (gravity, oral intubation, inadequate sucking) in preterm infants [4, 5, 37, 38]. Furthermore, maxillary measurements are widely used and, with appropriate correction factors, can be applied to the mandible.

Crown length measurements were performed by a single trained investigator, which may limit generalisability. However, intrarater reliability was excellent, supporting measurement consistency.

In addition, crown eruption was assessed on digitised plaster casts rather than by direct clinical observation or direct intraoral scans, representing an indirect but high-resolution reconstruction of in vivo dental development.

The timing of the first examination differed between term and preterm infants. Although analyses based on corrected age partly address differences in developmental maturity, the later and more variable timing of the first examination in preterm infants may have influenced the estimation of early eruption onset.

While major congenital or systemic conditions were excluded by the trial protocol, the present analysis did not include additional adjustment for neonatal health status or neonatal complications. These factors should be considered when interpreting the model-based eruption estimates and their generalisability.

Although longitudinal impressions were available, the data did not constitute a dense continuous eruption record for each child. Consequently, estimates of eruption timing and velocity depend on the assumed asymptotic model, and children with more available impressions may have contributed proportionally more information to the fitted population-level curves.

The actual model of eruption velocity remain speculative, as there are no comparative studies to date and data concerning the gingival thickness in such young children is not available. However, comparison with the related and well-studied growth rates of mineralisation and root length suggests a realistic eruption rate.

## Conclusions

Primary tooth eruption was largely comparable between preterm and term infants, with a statistically significant difference observed for canines when assessed by chronological age. Model-based trajectories of crown exposure on serial maxillary casts suggested that the onset of eruption preceded commonly reported clinical eruption ages by about 6.2 months in preterm infants and 7.7 months in term infants. The agreement between literature eruption ages and the model-estimated half-eruption time point indicates that many clinical reports likely reflect a mid-eruption stage rather than eruption onset. Future studies should therefore define the eruption criterion used and, where possible, document visible crown length at first observation to improve comparability and enable more consistent estimation of eruption onset, particularly when relating eruption to transient clinical symptoms. Eruption velocity followed a non-linear pattern comparable in magnitude to other dental growth processes.

## Data Availability

The data presented in this study are available on reasonable request from the corresponding author.
